# Simulated weightbearing computed tomography for verification of radiographic staging of varus ankle osteoarthritis: a cross-sectional study

**DOI:** 10.1186/s12891-021-04618-6

**Published:** 2021-08-28

**Authors:** Kiyonori Tomiwa, Yasuhito Tanaka, Hiroaki Kurokawa, Kunihiko Kadono, Akira Taniguchi, Korakot Maliwankul

**Affiliations:** 1 Department of Orthopedic Surgery, Nara Medical University, 840 Shijo, Kashihara, Nara, 634-8522 Japan; 2 Department of Orthopedics, Prince of Songkla University, 15 Karnjanavanich Road, Hat Yai, Songkhla 90110 Thailand

**Keywords:** Ankle osteoarthritis, varus, Stage classification, Weight-bearing computed tomography

## Abstract

**Background:**

Varus ankle osteoarthritis is classified using only weightbearing anteroposterior ankle radiographs; however, sagittal ankle alignment may also affect the position and extent of joint space obliteration. We hypothesized that the sagittal alignment of the ankle may also affect the position and extent of joint space obliteration visible on the coronal section; therefore, we identified the sites of joint space obliteration in patients with stage 3 varus ankle osteoarthritis for comparison with the sites observed on simulated weightbearing computed tomography and investigated the effects of anterior and posterior ankle subluxation.

**Methods:**

Simulated weightbearing computed tomography scans of 83 ft with varus ankle osteoarthritis (26 stage 3a, 57 stage 3b) were performed to check for joint space obliteration in the ankle. Further classification as exhibiting either anterior, posterior, or no subluxation on weightbearing lateral radiographs was performed.

**Results:**

Anterior, posterior, and no subluxation was seen in 5, 9, and 12 ankles among the 26 classified as stage 3a, respectively, and in 22, 12, and 23 ankles among the 57 classified as stage 3b, respectively. The mean tibial lateral surface angle on weightbearing lateral radiographs in stage 3a ankles was 75.6, 83.3, and 80.3 degrees in the anterior, posterior, and no subluxation groups, respectively; and 75.5, 86.6, and 82.7 degrees in stage 3b ankles (*p < .05*). In stage 3b ankles, widespread joint space obliteration was observed at the anterior distal articular surface of the tibia in all 22 ankles with anterior subluxation and at the posterior distal articular surface of the tibia in all 12 ankles with posterior subluxation.

**Conclusions:**

Simulated weightbearing computed tomography revealed joint space obliteration at the anterior distal articular surface of the tibia in stage 3b ankles with anterior subluxation and at the posterior side in stage 3a and 3b ankles with posterior subluxation. In some patients with stage 3 varus ankle osteoarthritis, the obliteration of the joint space is difficult to evaluate accurately using only weightbearing anteroposterior radiographs; weightbearing lateral radiographs should also be performed.

## Background

Ankle osteoarthritis is classified as either varus or valgus according to the joint alignment, with the varus type being more frequent [1]. Recent advances in operative devices and improvements in operative procedures for varus ankle osteoarthritis have led to osteotomy being commonly performed. Osteoarthritis staging is important for operative indications and selection of operative procedures. One widely used grading system for varus ankle osteoarthritis is the Takakura-Tanaka classification [2, 3], which classifies the stage from 1 to 4 based on weightbearing anteroposterior (AP) ankle radiographs. Studies of the postoperative outcomes of low tibial osteotomy have led to further subclassification of stage 3 (obliteration of the talocrural joint space) into stage 3a (obliteration of the talocrural joint space limited to the articular surface of the medial malleolus) and stage 3b (joint space obliteration also affecting the roof of the talar dome).

In general, low tibial osteotomy is indicated up to stage 3a [3]. An accurate understanding of the differences between the pathologies of stage 3a and stage 3b is important when considering the indications for osteotomy [3]. Thus far, joint space obliteration has been classified entirely on the basis of weightbearing AP ankle radiographs. However, three-dimensional (3D) studies are required to verify which parts of the articular surfaces are affected. Varus ankle osteoarthritis includes ankles in which the position of the talus is subluxated either anteriorly or posteriorly with regard to the distal articular surface of the tibia on weightbearing lateral ankle radiographs. We hypothesized that the sagittal alignment of the ankle may also affect the position and extent of joint space obliteration visible in the coronal section.

Our objectives were to accurately identify sites of joint space obliteration in patients with stage 3a or 3b varus ankle osteoarthritis diagnosed on weightbearing AP ankle radiographs. We hypothesized that the sagittal alignment of the ankle may also affect the position and extent of joint space obliteration visible on the coronal section.

## Methods

Between 2011 and 2015, 190 patients underwent surgery for varus ankle osteoarthritis at our hospital. Of these, 110 were diagnosed with stage 3a or 3b varus ankle osteoarthritis, and simulated weightbearing computed tomography (SW-CT) scans were performed on 92 ankles after obtaining written informed consent for the imaging test. The final study population comprised 26 ankles from 24 patients classified as stage 3a and 57 ankles from 53 patients classified as stage 3b, totaling 83 ankles from 77 patients.

**Fig. 1:**
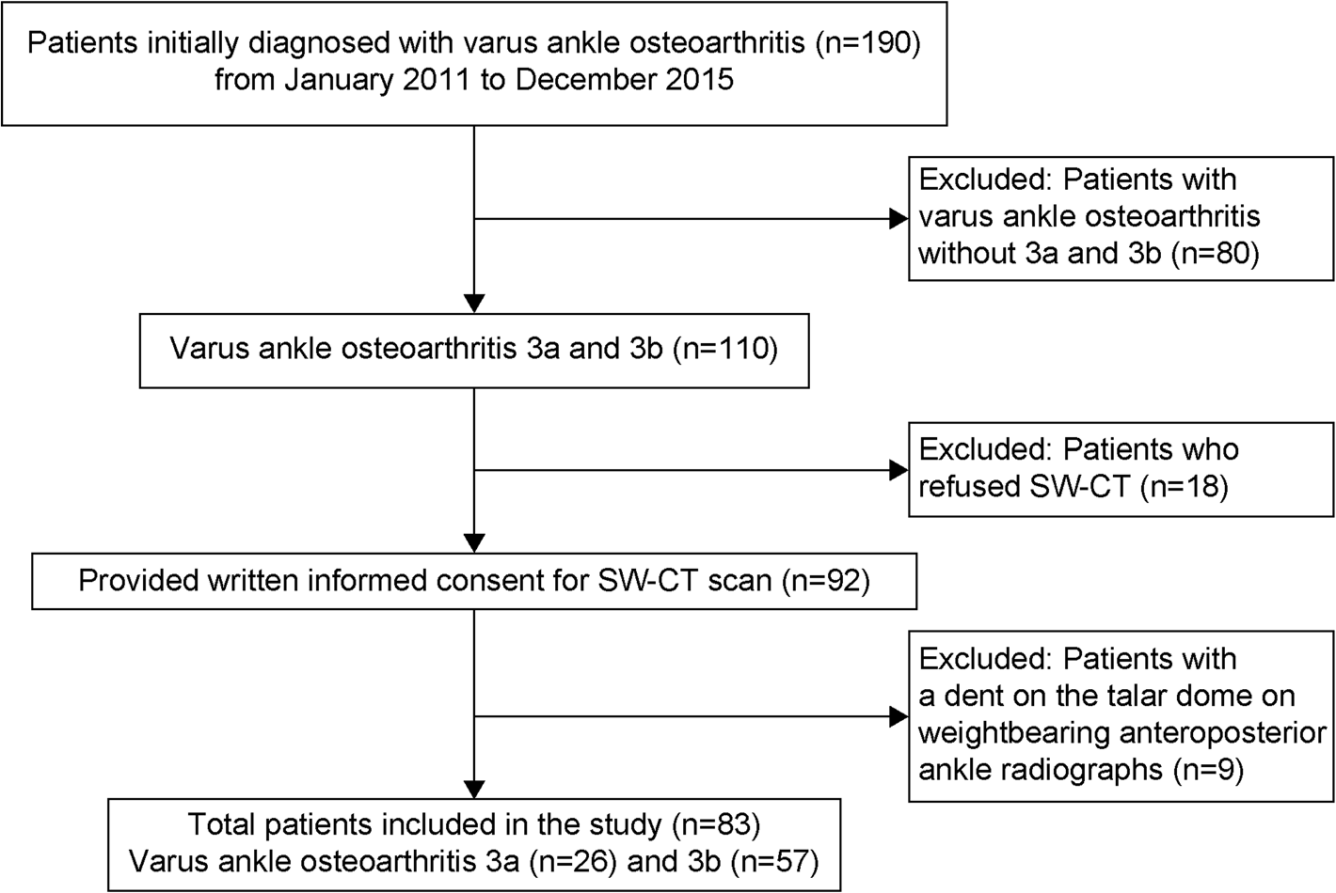
Flowchart of the patient selection process

Figure 1 shows a flowchart of the patient selection process. Staging was performed by two specialist foot and ankle surgeons using weightbearing AP ankle radiographs; if their opinions concurred, this was considered the correct stage. If they disagreed, a third foot and ankle surgeon verified their assessments, and the majority’s opinion was adopted.

In this study, the reason to include patients classified only as stage 3a and 3b was to evaluate the pathology of pure varus osteoarthritis. This is because joint space obliteration is easy to assess, and SW-CT assessment would be more difficult at stage 1 or 2 when joint space obliteration has not occurred. Stage 4 patients were excluded because, according to Hayashi et al. [4], stage 4 encompasses different conditions, and such patients may not reflect the pathology of pure varus osteoarthritis.

Table 1 shows the demographic data of study participants. We retrospectively compared the findings of weightbearing radiographs and SW-CT scans of these feet, as described below, and investigated the extent of the effect of anterior and posterior ankle subluxations.

**Table 1:** Patient characteristics

	Overall (3a + 3b)	3a	3b
N	83 (100%)	26 (31%)	57 (69%)
Mean age [min-max]	67.7 [26–86]	66.9 [57–75]	68.1 [26–86]
Sex			
Male	10 (12%)	3 (30%)	7 (70%)
Female	73 (88%)	23 (32%)	50 (68%)
Mean height (cm) [min-max]	153.3 [138–188]	154.5 [142–175]	152.8 [138–188]
Mean weight (kg) [min-max]	57.4 [36–89.5]	60.9 [46–89.5]	55.7 [36–83.1]
Mean BMI (kg/cm^2^) [min-max]	24.3 [17.2–34.7]	25.5 [19.9–33.4]	23.9 [17.2–34.7]
Left/right	39/44	15/11	24/33
Bilateral cases	6	2	4

*BMI* Body mass index, *min* Minimum, *max* Maximum

### Radiographic method and measurements

**Fig. 2:**
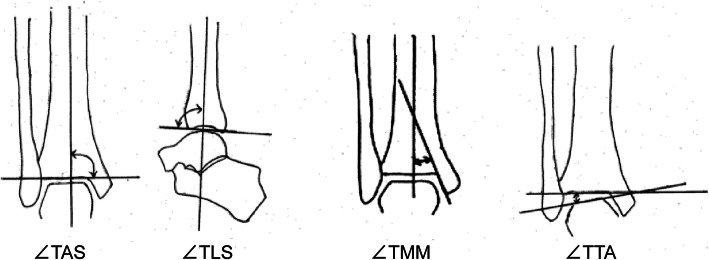
Radiological measurements of the ankle. Tibial articular surface angle (∠TAS). Tibial lateral surface angle (∠TLS). Tibial medial malleolus angle (∠TMM). Talar tilt angle (∠TTA)

Weightbearing AP and lateral radiographs of the ankle were performed. During this process, the patient held onto a handrail and stood on one leg while the X-ray beam was focused on the center of the ankle, and radiographs were taken from a distance of 1 m. Lateral radiographs were taken with the beam directed from the medial to the lateral side. Ankle joint alignments were measured as shown in Fig. 2.

We used the tibial lateral surface angle (∠TLS) as a measure of sagittal alignment to investigate the effect of sagittal alignment as a cause of anterior subluxation of the talus with regard to the distal articular surface of the tibia.

### Assessment and classification of talar subluxation in the sagittal plane

As there are no definite assessment criteria for ankle subluxation, we measured the positional relationship between the tibia and the talus using the same locations as used by Hackenbruch et al. [5] to measure the anterior talar translation of the ankle; this required measuring the shortest distance from the posterior margin of the articular surface of the tibia to the articular surface of the talar dome (a) on weightbearing lateral radiographs.

**Fig. 3:**
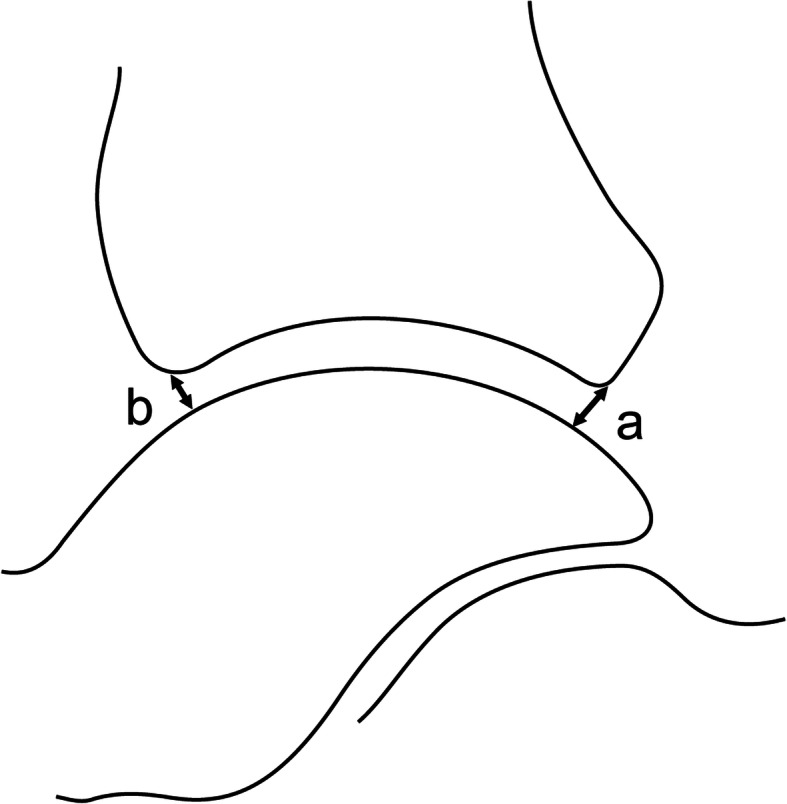
Subluxation of the talus. A difference between these two measurements of less than 1 mm was classified as no subluxation, while a difference of 1 mm or more was classified as subluxation. (a - b ≥ 1) was classified as anterior subluxation. (b - a ≥ 1) was classified as posterior subluxation. **a** Distance from the posterior margin of the tibia articular surface to the talar dome articular surface. **b** Distance from the anterior margin of the tibia articular surface to the talar dome articular surface

We similarly measured the shortest distance from the anterior margin of the articular surface of the tibia to the articular surface of the talar dome (b). A difference between these two measurements of less than 1 mm was classified as no subluxation, while a difference of 1 mm or more was classified as subluxation. In patients with subluxation, if the distance from the posterior margin of the articular surface of the tibia to the articular surface of the talar dome (a) was 1 mm greater than the distance from the anterior margin of the articular surface of the tibia to the articular surface of the talar dome (b) (a - b ≥ 1), this was classified as anterior subluxation. Conversely, if the distance from the anterior margin of the articular surface of the tibia to the articular surface of the talar dome (b) was 1 mm greater than the distance from the posterior margin of the articular surface of the tibia to the articular surface of the talar dome (a) (b - a ≥ 1), this was classified as posterior subluxation (Fig. 3).

Patients were divided into subgroups according to the relative position of the talus with regard to the tibia based on weightbearing lateral ankle radiographs. Those with anterior subluxation of the talus were classified as the anterior subluxation group, those with posterior subluxation of the talus as the posterior subluxation group, and those with no obvious subluxation as the no subluxation group.

### SW-CT scanning and assessment

**Fig. 4:**
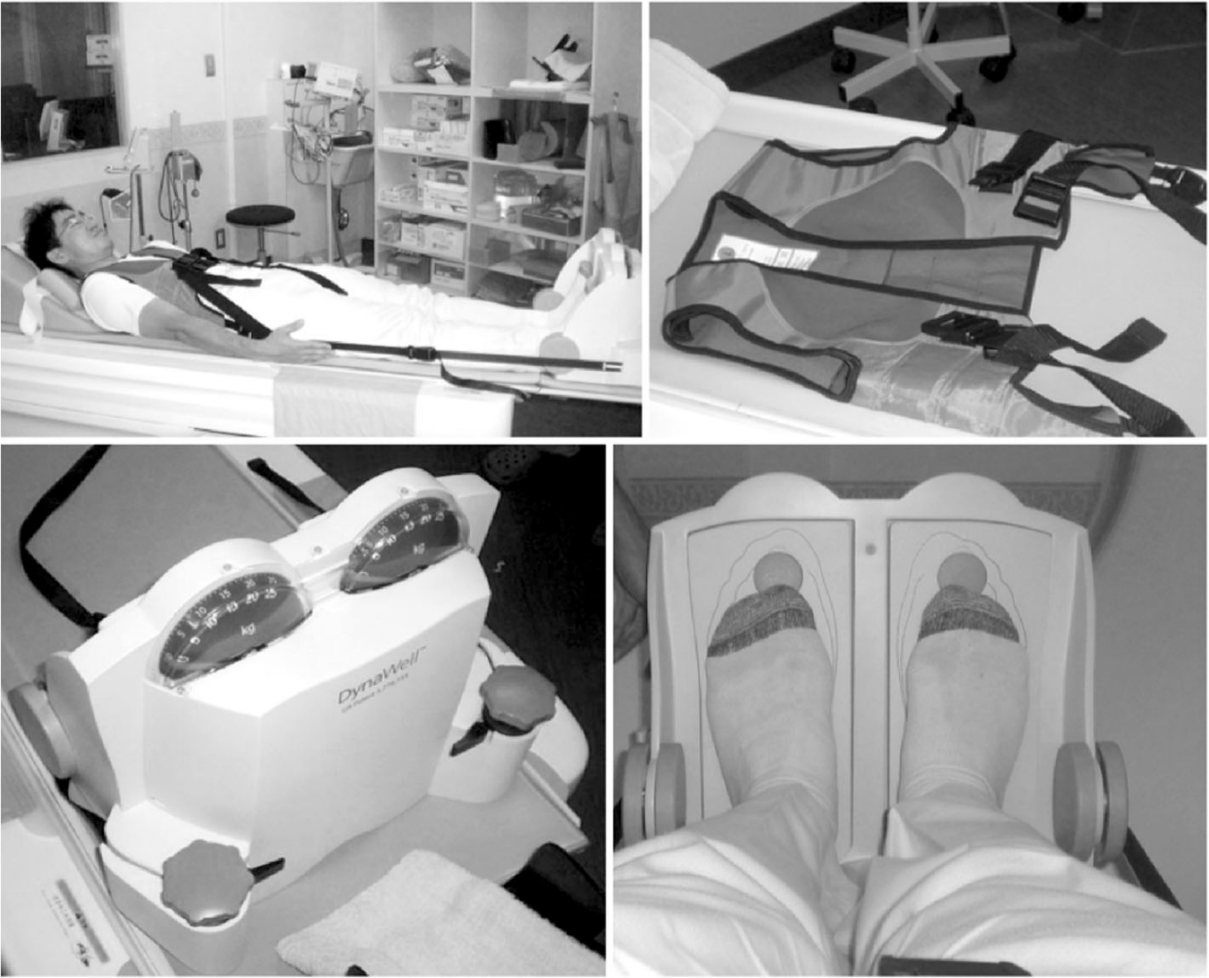
Experimental arrangement in the simulated weightbearing computed tomography. All patients wore a special vest, straps, force meter, and footplate and were scanned with a simulated weightbearing of 30 kgf. The individual shown in the image is an author of this study (KT)

**Fig. 5:**
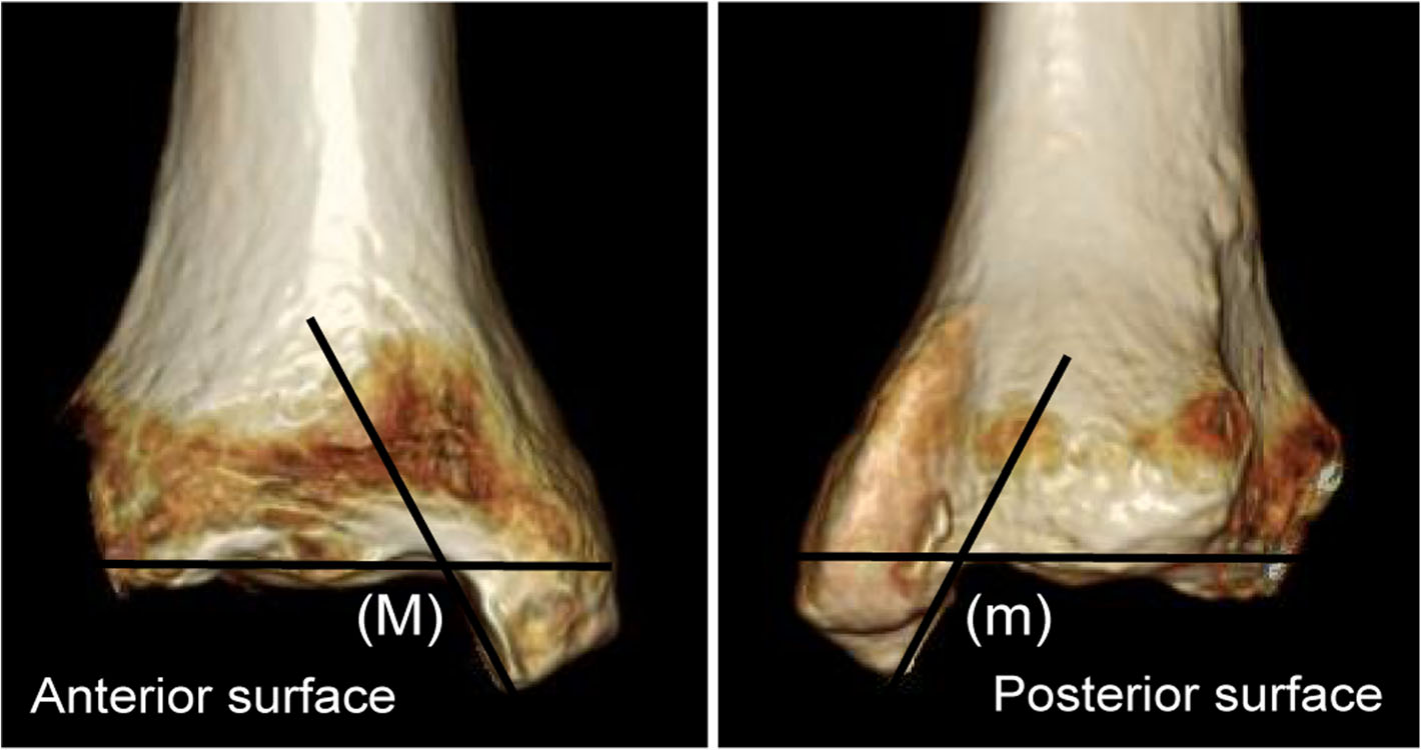
Anterior and posterior three-dimensional computed tomography images of the distal articular surface . M: Intersection point of the tangent of the distal anterior articular surfaces and the malleolar articular surface on the anterior sides of the tibia, respectively. m: Intersection point of the tangent of the distal posterior articular surfaces and the malleolar articular surface on the posterior sides of the tibia, respectively

**Fig. 6:**
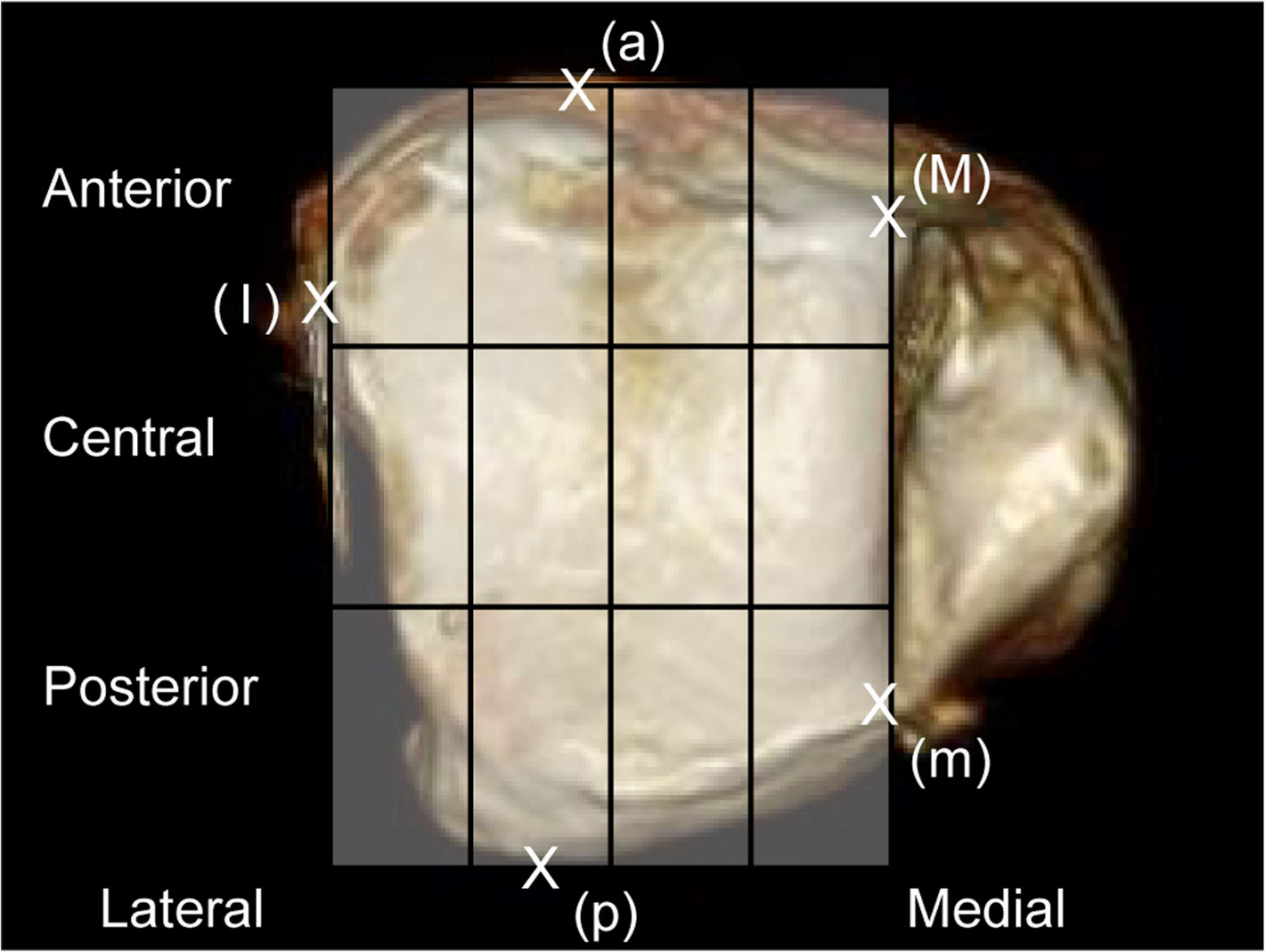
Distal articular surface three-dimensional computed tomography images of the tibia. Assessment was performed using a rectangle connecting the line Mm, anterior border (a), posterior border (p), and lateral border (l). The rectangle was divided into three sections (anterior, central, and posterior) anteroposteriorly and four sections mediolaterally

CT scans were obtained using an OPTIMA660 device (General Electric, Boston, MA). The device used for simulated weightbearing was a DynaWell L-Spine (DynaWell Inc., Las Vegas, NV). This was used to reproduce the sagittal alignment in the standing position while the patient was supine during a lumbar CT or magnetic resonance imaging (MRI), with patients fitted with a special vest, straps, force meter, and footplate [6]. All patients were scanned with a simulated weightbearing of 30 kgf (Fig. 4). Assessment of the distal articular surface of the tibia was performed using the medial border (Mm), which is a straight line connecting the intersection points of the tangents of the distal and malleolar articular surfaces on the anterior and posterior surfaces of the tibia (Fig. 5). The assessment also utilized the rectangle connecting the anterior border (a), posterior border (p), lateral border (l), and medial border (Mm) of the distal articular surface of the tibia (Fig. 6).

**Fig. 7:**
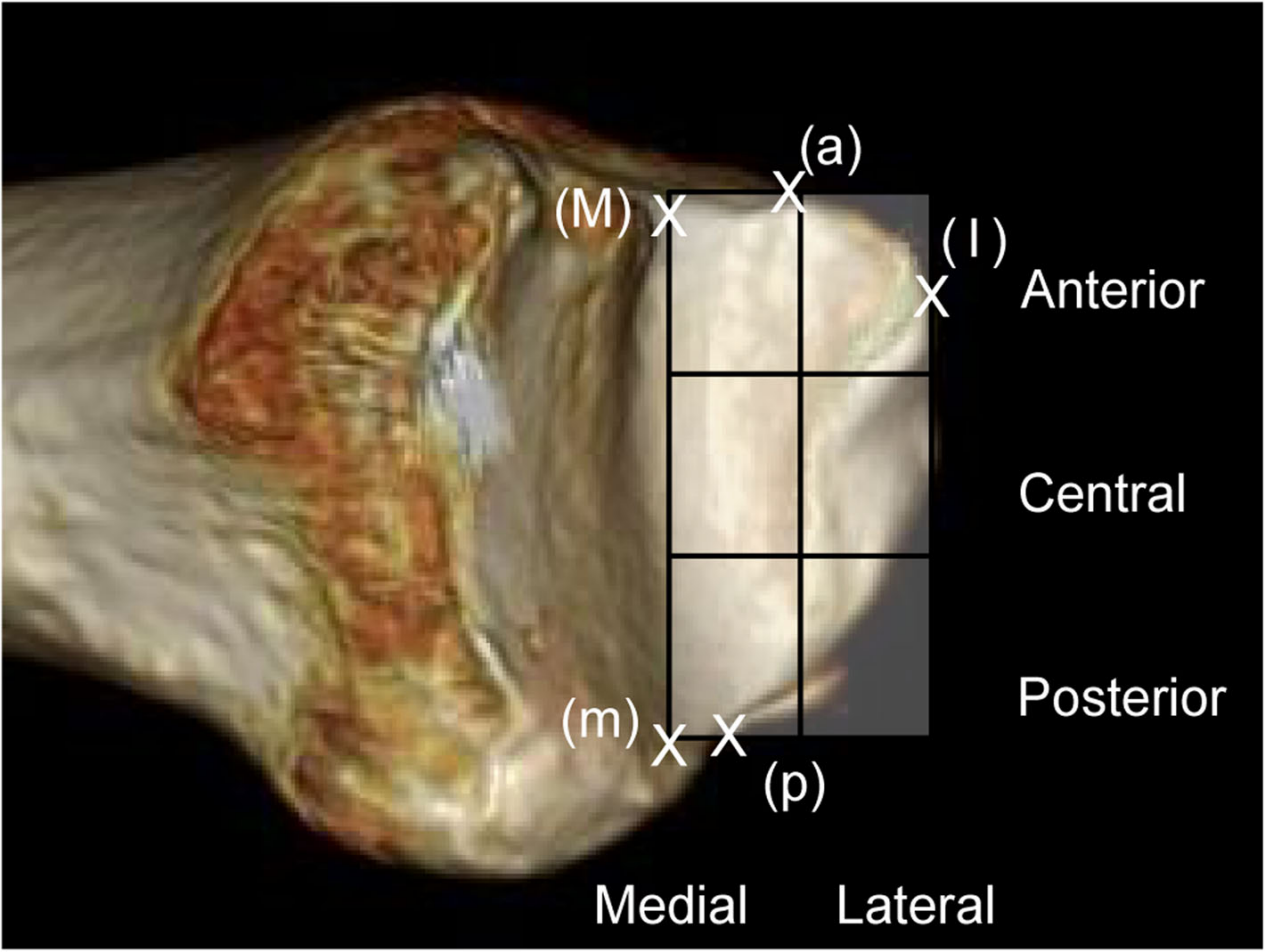
Malleolar surface image of the tibia with 3D CT. Assessment was performed using the line Mm, anterior border (a), posterior border (p), and distal portion (lateral border) (l). The rectangle was divided into three sections (anterior, central, and posterior) anteroposteriorly and two sections mediolaterally

Assessment of the malleolar articular surface was also performed using the medial border (Mm), which is a straight line connecting the intersection points of the tangents of the distal and malleolar articular surfaces on the anterior and posterior surfaces of the tibia (Fig. 5). The assessment also utilized the rectangle connecting the anterior border (a), posterior border (p), lateral border (l), and medial border (Mm) of the malleolar articular surface of the tibia (Fig. 7).

The distal articular surface of the tibia was divided into three sections anteroposteriorly (anterior, central, and posterior) and four sections mediolaterally, creating a 12-square grid (Fig. 6). Joint space obliteration was assessed in each square grid on the CT scan. For each of the four-square grids in each (anterior, central and posterior) row, moving from the medial to the lateral side, we assessed and recorded which row had maximum damage in each case. In grids that included parts where the joint surface was not visible, assessment was only performed on the parts with visible joint surface. However, if any joint space obliteration was seen in each grid, it was deemed that cartilage damage was observed in that particular grid. The row (anterior, central, or posterior) that contained the widest area of joint space obliteration was defined as the row with maximum damage. If two or more of the three rows had the same levels of damage, the SW-CT scans were reviewed, and the row in which the damage extended laterally for the greatest distance was considered the row with maximum damage. The malleolar articular surface of the tibia on the distal articular surface of the tibia was divided anteroposteriorly into three rows (anterior, central, and posterior) and mediolaterally into two to produce a six-square grid and was assessed in a similar manner (Fig. 7).

This study was approved by Nara Medical University Ethics Committee, Japan (IRB no. 848). Written informed consent in accordance with the Declaration of Helsinki was obtained from all study participants.

### Image analysis

All radiograph and CT image data were obtained using the Digital Imaging and Communications in Medicine standard. SYNAPSE VINCENT Ver5.1 (Fujifilm, Tokyo, Japan) was used for all image measurements and analyses.

### Statistical analysis

A t-test was used for all statistical comparisons between ankles classified as stage 3a and stage 3b. EZR (Easy R) on R commander (EZR Version 1.41, Y. Kanda, Saitama, Japan) was used for all statistical analyses (*p* < 0.05 was considered significant) [7].

## Results

The intra-rater and inter-rater reliability was excellent for all measurements. The ICCs are presented in Table 2.

**Table 2:** Intra- and inter-rater reliability for distance and angle measurements

	Reliability measurement	Rater 1	Rater 2	Rater 3
(a – b) or (b – a)	intra-rater	0.964	0.955	0.989
	inter-rater	0.987		
∠TAS	intra-rater	0.963	0.917	0.883
	inter-rater	0.955		
∠TLS	intra-rater	0.981	0.964	0.985
	inter-rater	0.988		
∠TMM	intra-rater	0.997	0.993	0.997
	inter-rater	0.990		
∠TTA	intra-rater	0.980	0.989	0.991
	inter-rater	0.989		

a: distance from the posterior margin of the articular surface of the tibia to the articular surface of the talar dome

b: distance from the anterior margin of the articular surface of the tibia to the articular surface of the talar dome

∠*TAS*: Tibial articular surface angle

∠*TLS*: Tibial lateral surface angle

∠*TMM*: Tibial medial malleolus angle

∠*TTA*: Talar tilt angle

**Fig. 8:**
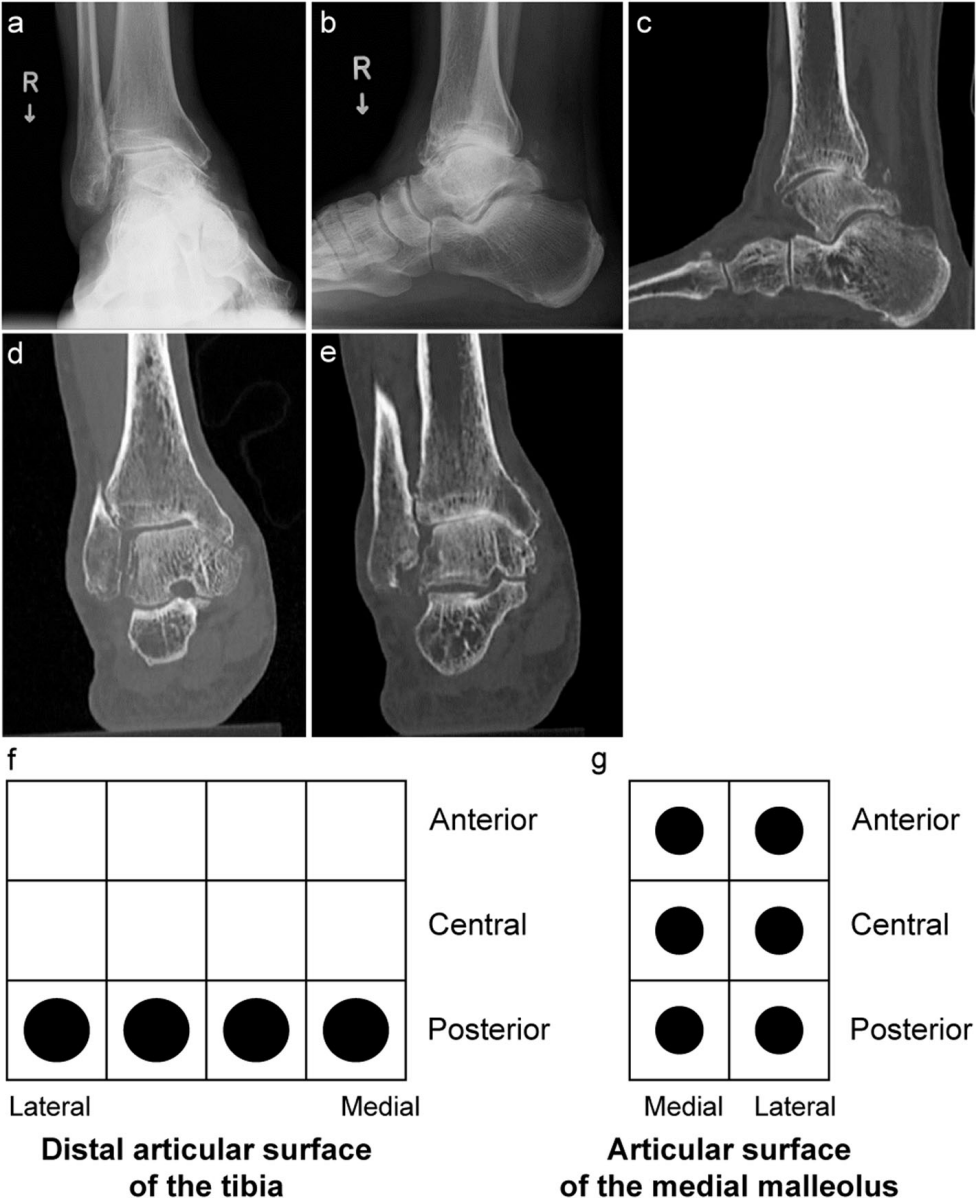
Stage 3a osteoarthritis of the right ankle in a 65-year-old man. **a** Stage 3a osteoarthritis on weightbearing frontal radiograph. **b** Posterior subluxation on weightbearing lateral radiograph. **c** Joint space narrowing on sagittal simulated weightbearing computed tomography (SW-CT). **d**, **e** On coronal SW-CT, evidence of joint space obliteration is not widespread at the anterior distal articular surface of the tibia (**d**), but widespread joint obliteration was evident posteriorly (**e**). **f** The entire posterior row was mapped in the distal articular surface of the tibia with a 12-square grid by mapping result of SW-CT as in Fig. 8d, e. g: All anterior, central, and posterior rows were mapped in the articular surface of the medial malleolus with a 6-square grid by mapping result of SW-CT as in Fig. 8d, e

Of the 26 ankles classified as stage 3a, 5, 9, and 12 showed anterior, posterior, and no subluxation, respectively. On SW-CT, 5 out of the 9 ankles with posterior subluxation exhibited joint space obliteration at the posterior distal articular surface of the tibia, 2 of which showed widespread joint space obliteration (Fig. 8). Among the 12 ankles with no subluxation, 2 exhibited joint space obliteration at the posterior distal articular surface of the tibia.

**Fig. 9:**
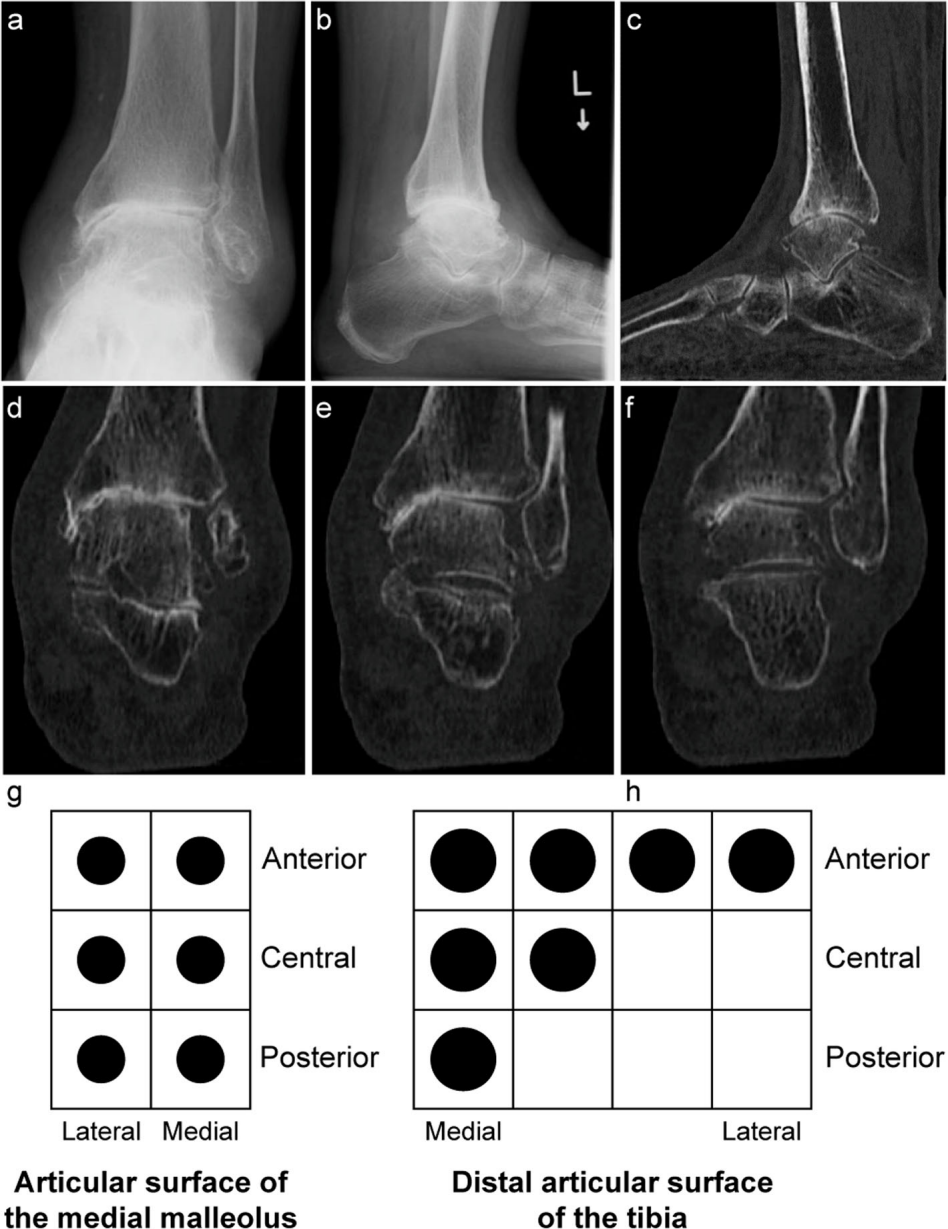
Stage 3b osteoarthritis of the left ankle in a 74-year-old woman. **a** Stage 3b osteoarthritis on weightbearing frontal radiograph. **b** Anterior subluxation on weightbearing lateral radiograph. **c** Joint space obliteration on sagittal simulated weightbearing computed tomography (SW-CT). **d**, **e**, **f** On coronal SW-CT, joint space obliteration is widespread at the anterior distal articular surface of the tibia (d) but is narrower across the center (e) posteriorly (f). **g** All sections were mapped in the articular surface of the medial malleolus, by mapping result of SW-CT as in Fig. 9d, e, f. **h** Four sections on the anterior row, three sections on the central row, and one section on the posterior row were mapped in the distal tibia articular surface by mapping result of SW-CT as in Fig. 9d, e, f

Of the 57 ankles classified as stage 3b, 22, 12, and 23 exhibited anterior, posterior, and no subluxation, respectively. With regard to the direction of subluxation and the area of maximum damage on SW-CT, all 22 anteriorly subluxated ankles had greater damage in the anterior row (Fig. 9). Similarly, all 12 posteriorly subluxated ankles had greater damage in the posterior row. Among the 23 ankles with no subluxation, the row with the maximum damage was the central row in 3 ankles. In the remaining 20 ankles, the row with the maximum damage was the anterior row in 11 ankles and the posterior row in 9 ankles (Table 3).

**Table 3:** Direction of subluxation and row with maximum damage on the distal articular surface

Stage	Subluxation	Row with maximum damage on the distal articular surface
3a	anteriorly 5	none 5
	posteriorly 9	posterior 5, none 4
	none 12	posterior 2, none 10
3b	anteriorly 22	anterior 22
	posteriorly 12	posterior 12
	none 23	anterior 11, posterior 9, central 3

With regard to alignment, no significant difference was found between ankles classified as stages 3a and 3b in terms of the tibial surface angle (∠TAS) or ∠TLS, but significant differences were noted between the tibial medial malleolus angle (∠TMM) and the talar tilt angle (∠TTA) (*p* < 0.05, Table 4).

**Table 4:** Ankle alignment. t-test was used for statistical comparisons between stage 3a and stage 3b

	Overall (3a + 3b) Mean ± SD	3a Mean ± SD	3b Mean ± SD	*P*-value
∠TAS	82.9 ± 3.54	83.9 ± 2.13	82.5 ± 3.98	***P*** **= 0.074**
∠TLS	80.6 ± 5.60	80.4 ± 4.01	80.7 ± 6.26	***P*** **= 0.815**
∠TMM	54.1 ± 13.1	45.0 ± 9.36	58.3 ± 12.5	***P*** **< 0.001**
∠TTA	10.4 ± 6.11	6.73 ± 4.49	12.1 ± 6.04	***P*** **< 0.001**

Values are presented as mean ± standard deviation (SD)

∠*TAS*: Tibial articular surface angle

∠*TLS*: Tibial lateral surface angle

∠*TMM*: Tibial medial malleolus angle

∠*TTA*: Talar tilt angle

**Fig. 10:**
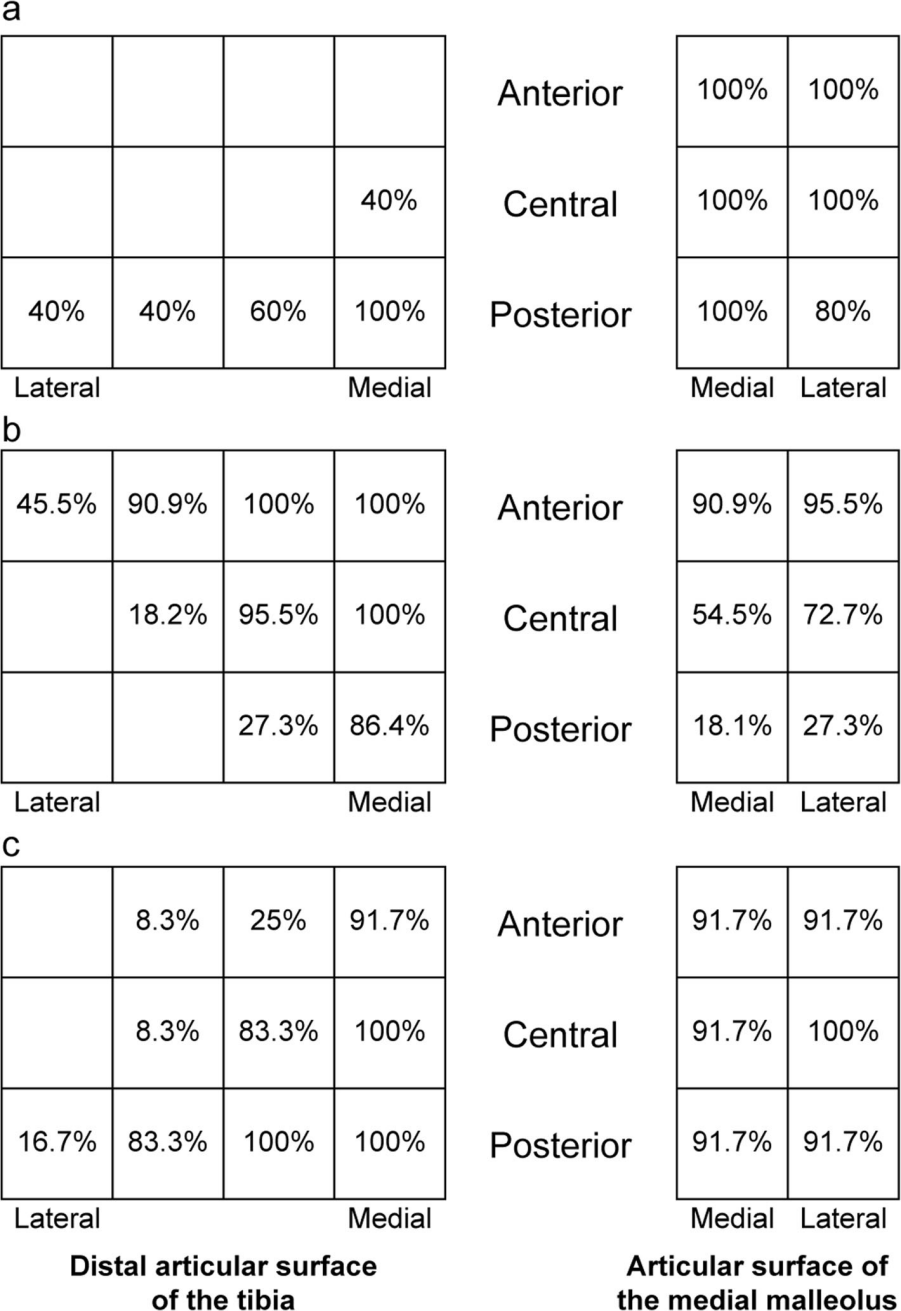
Distribution of the joint space obliteration owing to talar subluxation in ankle osteoarthritis. **a** Damage rates to the distal articular surface of the tibia and articular surface of the medial malleolus in stage 3a osteoarthritis and posterior subluxation (*n* = 5). The row with maximum damage of the distal articular surface was the posterior side, defined as the talar posterior subluxation. **b** Damage rates to the distal articular surface of the tibia and articular surface of the medial malleolus in stage 3b osteoarthritis and anterior subluxation (*n* = 22). The row with maximum damage of the distal articular surface was the anterior side, defined as the talar anterior subluxation. **c** Damage rates to the distal articular surface of the tibia and articular surface of the medial malleolus in stage 3b with posterior subluxation (*n* = 12). The row with maximum damage of the distal articular surface was the posterior side, defined as the talar posterior subluxation

Figure 10 shows the distribution of joint space obliteration at the distal articular surface of the tibia in ankles classified as stages 3a and 3b. This figure was standardized for the distal articular surface of the tibia and the medial articular surface of the tibia of the right ankle when looking upward from the caudal side and reversed for the left foot. In most ankles, the row with maximum damage was on the same side as the direction of the talar subluxation, whether this was anterior or posterior, and its extent on the sagittal section also increased. In all cases, damage occurred in one of the rows on the medial side, and the rate of damage decreased in the more lateral rows.

In ankles classified as stage 3a, the mean ∠TLS was 75.6, 80.3, and 83.3 degrees for those with anterior subluxation, no subluxation and posterior subluxation respectively, also differing significantly among the three groups (*p* < 0.05*)*.

In ankles with stage 3b, the mean ∠TLS was 75.5, 82.7 and 86.6 degrees in ankles with anterior subluxation, no subluxation, and posterior subluxation, respectively, differing significantly between the three groups *(p* < 0.05*)* (Table 5).

**Table 5:** Comparisons of the ankle ∠TLS among the three groups for stages 3a and 3b

Type	∠TLS Mean ± SD	Comparison	*P*-value
3a Anterior subluxation	75.6 ± 4.67	vs. no subluxation	***P*** **= 0.022**
3a No subluxation	80.3 ± 2.83	vs. posterior subluxation	***P*** **= 0.017**
3a Posterior subluxation	83.3 ± 2.06	vs. anterior subluxation	***P*** **< 0.001**
3b Anterior subluxation	75.5 ± 4.60	vs. no subluxation	***P*** **< 0.001**
3b No subluxation	82.7 ± 4.90	vs. posterior subluxation	***P*** **= 0.026**
3b Posterior subluxation	86.6 ± 3.32	vs. anterior subluxation	***P*** **< 0.001**

*SD* Standard deviation

## Discussion

In this study, we found that the site of damage to the distal articular surface of the tibia in varus ankle osteoarthritis is located either anteriorly or posteriorly according to the severity of anterior or posterior subluxation of the talus. This cannot be diagnosed by the weightbearing AP ankle radiographs conventionally used for osteoarthritis. In our study, we carried out a detailed investigation of the state of damage to the distal articular surface of the tibia and the articular surface of the medial malleolus by simulated weightbearing in the standing position, following reports of lumbar CT and MRI scans simulating standing weightbearing [6].

In the Takakura-Tanaka classification of varus ankle osteoarthritis, the stage is classified in terms of the state of the joint space between the talus, medial malleolus, and roof of the talar dome, using weightbearing AP radiographs of the ankle [2, 3]. However, no classification has used weightbearing lateral radiographs of the ankle yet, and the site of cartilage damage in the anteroposterior orientation has not been addressed. Additionally, few studies on anteroposterior cartilage using weightbearing lateral radiographs of the ankle have been performed. In terms of the etiology of ankle osteoarthritis, on the AP radiograph, when the distal tibial joint surface is in varus, the loading shifts to the medial side because of increased stress on that area. On the lateral radiograph, anterior loading is increased by the anterior opening of the joint [2].

In our study, we investigated anterior and posterior subluxation in stage 3 varus ankle osteoarthritis and found significant differences in the ∠TLS between ankles exhibiting anterior, posterior, and no subluxation. The anterior tilt of the ∠TLS tended to be smaller in patients with anterior subluxation of the talus and greater in those with posterior subluxation. This was found in both stage 3a and 3b ankles. When we compared the mapped sites of joint space obliteration on the distal articular surface of the tibia between stages 3a and 3b ankles and between anterior and posterior subluxation, joint space obliteration was clearly greater on the posterior side in stage 3a ankles with posterior subluxation of the talus. By contrast, in stage 3b ankles with anterior subluxation, joint space obliteration was clearly greater on the anterior side. Similarly, in cases of posterior subluxation, joint space obliteration was clearly greater on the posterior side. This was also true for ankles with posterior subluxation. We considered that when the ∠TLS is smaller and there is anterior opening of the joint, the talus is subluxated anteriorly, causing arthritis of the distal articular surface of the tibia at this site. Conversely, when the ∠TLS is greater and there is posterior opening of the joint, the talus is subluxated posteriorly, leading to arthritis mainly in this part of the distal articular surface of the tibia.

Regarding the alignment between the tibia and the talus in the lateral plane, Tochigi et al. [8, 9] reported that anterior or posterior subluxation of the talus with regard to the tibia places the joint under stress, causing ankle osteoarthritis. They also found that in 32 osteoarthritic ankles, 5 and 11 exhibited anterior and posterior subluxation, respectively, of the talus with regard to the tibial articular surface on weightbearing lateral radiographs and reported that those patients with subluxation had end-stage ankle osteoarthritis. Veljkovic et al. [10] measured the position of the center of the talar dome in relation to the tibial axis on images from 82 patients without ankle osteoarthritis. Magerkurth et al. [11] compared 52 patients with chronic ankle instability and 52 healthy individuals and found that the position of the talar center of rotation with regard to the tibial axis was significantly anteriorized in patients with chronic ankle instability. Talar subluxation should be assessed on the basis of the relative position of the talus to the articular surface of the tibia, but currently, no study has reported the association of this direction with areas of damage to the distal articular surface of the tibia. Here, we measured the distances between the articular surface of the talar dome and the anterior and posterior margins of the articular surface of the tibia on weightbearing lateral radiographs and used them as a new index to assess the relative position of the talus in relation to the articular surface of the tibia and define subluxation.

These findings indicated the feasibility of using weightbearing lateral radiographs to verify the location of progressive cartilage damage on the distal articular surface of the tibia in patients with stage 3a or 3b varus ankle osteoarthritis. This may be helpful for preoperative planning in an attempt to distribute the load to areas with fewer arthritic changes when performing osteotomy of the distal tibia to treat varus ankle osteoarthritis, potentially improving therapeutic outcomes.

In one patient with stage 3b ankle osteoarthritis and no subluxation, the area of greatest damage was on the posterior distal articular surface of the tibia, but there was no joint space obliteration at the posterior articular surface of the medial malleolus. In another patient with no subluxation, the area of greatest damage was on the anterior distal articular surface of the tibia, but there was no joint space obliteration at the anterior articular surface of the medial malleolus. These patterns of damage are difficult to explain in terms of our present analysis, and it may be necessary to include an assessment of factors such as talar rotation.

This study has some limitations. First, weightbearing radiography was performed with the patients standing, but the SW-CT scans were carried out while they were in a supine position, which does not constitute a full weightbearing CT scan. Second, in the simulations, all patients were subjected to the same 30 kgf weightbearing, raising the possibility that the actual extent of joint space obliteration may have been underestimated on the CT images. Third, although this study was a static analysis based on imaging, dynamic elements are also involved in joint space obliteration. Finally, we did not carry out quantitative assessments of other factors such as ankle instability.

In conclusion, in stage 3 varus ankle osteoarthritis, accurately evaluating not only weightbearing anteroposterior ankle radiographs but also lateral ankle radiographs before operation may help to choose the appropriate surgical intervention.

## Data Availability

The datasets used and/or analyzed during the current study will be made available from the corresponding author on reasonable request.

## Abbreviation

3D: Three-dimensional
AP: Anteroposterior
CT: Computed tomography
MRI: Magnetic resonance imaging
SW-CT: Simulated weightbearing computed tomography
∠TLS: Tibial lateral surface angle
∠TAS: Tibial surface angle
∠TMM: Tibial medial malleolus angle
∠TTA: Talar tilt angle
