# Correction to: Simulated weightbearing computed tomography for verification of radiographic staging of varus ankle osteoarthritis: a cross-sectional study

**DOI:** 10.1186/s12891-021-04710-x

**Published:** 2021-10-13

**Authors:** Kiyonori Tomiwa, Yasuhito Tanaka, Hiroaki Kurokawa, Kunihiko Kadono, Akira Taniguchi, Korakot Maliwankul

**Affiliations:** 1 Department of Orthopedic Surgery, Nara Medical University, 840 Shijo, Kashihara, Nara 634-8522 Japan; 2 Department of Orthopedics, Prince of Songkla University, 15 Karnjanavanich Road, Hat Yai, Songkhla, 90110 Thailand

**Correction to: BMC Musculoskelet Disord 22, 737 (2021)**

**https://doi.org/10.1186/s12891-021-04618-6**

Following the publication of the original article [1] the authors noticed that the errors pointed out in Table 3 were not implemented. The original article [1] has been updated.

Below is the correct Table 3.

**Table 3:** Direction of subluxation and row with maximum damage on the distal articular surface

Stage	Subluxation	Row with maximum damage on the distal articular surface
	anteriorly 5	none 5
3a	posteriorly 9	posterior 5, none 4
	none 12	posterior 2, none 10
	anteriorly 22	anterior 22
3b	posteriorly 12	posterior 12
	none 23	anterior 11, posterior 9, central 3
